# Stability and Antioxidant Activity of Semi-synthetic Derivatives of 4-Nerolidylcatechol

**DOI:** 10.3390/molecules18010178

**Published:** 2012-12-24

**Authors:** Emerson Silva Lima, Ana Cristina Silva Pinto, Karla Lagos Nogueira, Luiz Francisco Rocha e Silva, Patricia Danielle Oliveira de Almeida, Marne Carvalho de Vasconcellos, Francisco Celio Maia Chaves, Wanderli Pedro Tadei, Adrian Martin Pohlit

**Affiliations:** 1 Universidade Federal do Amazonas, Avenida General Rodrigo Otávio Jordão Ramos, 3000, 69077-000 Campus Universitário, Manaus, Amazonas, Brazil; E-Mails: eslima@ufam.edu.br (E.S.L.); patt_danielle@hotmail.com (P.D.O.A.); marne@ufam.edu.br (M.C.V.); 2 Instituto Nacional de Pesquisas da Amazônia, Avenida André Araújo, 2936, 69067-375 Aleixo, Manaus, Amazonas, Brazil; E-Mails: anacristinadsp@gmail.com (A.C.S.P.); klnogueira86@gmail.com (K.L.N.); luizrocha_silva@hotmail.com (L.F.R.S.); tadei@inpa.gov.br (W.P.T.); 3 Embrapa Amazônia Ocidental, Rodovia AM-010, Km 29, Zona Rural, 69010-970 Caixa Postal 319 Manaus, Amazonas, Brazil; E-Mail: celio.chaves@cpaa.embrapa.br

**Keywords:** ABTS, antioxidants, bioactivity, cytotoxicity, DPPH, natural product derivatives, 4-NC, *Piper peltatum*, radical scavenger activity, stability

## Abstract

4-nerolidylcatechol (4-NC) is an unstable natural product that exhibits important antioxidant, anti-inflammatory and other properties. It is readily obtainable on a multi-gram scale through straightforward solvent extraction of the roots of cultivated *Piper peltatum* or *P. umbellatum*, followed by column chromatography on the resulting extract. Semi-synthetic derivatives of 4-NC with one or two substituent groups (methyl, acetyl, benzyl, benzoyl) on the O atoms have been introduced that have increased stability compared to 4-NC and significant *in vitro* inhibitory activity against the human malaria parasite *Plasmodium falciparum*. Antioxidant and anti-inflammatory properties may be important for the antiplasmodial mode of action of 4-NC derivatives. Thus, we decided to investigate the antioxidant properties, cytotoxicity and stability of 4-NC derivatives as a means to explore the potential utility of these compounds. 4-NC showed high antioxidant activity in the DPPH and ABTS assays and in 3T3-L1 cells (mouse embryonic fibroblast), however 4-NC was more cytotoxic (IC_50_ = 31.4 µM) and more unstable than its derivatives and lost more than 80% of its antioxidant activity upon storage in solution at −20 °C for 30 days. DMSO solutions of mono-*O*-substituted derivatives of 4-NC exhibited antioxidant activity and radical scavenging activity in the DPPH and ABTS assays that was comparable to that of BHA and BHT. In the cell-based antioxidant model, most DMSO solutions of derivatives of 4-NC were less active on day 1 than 4-NC, quercetin and BHA and more active antioxidants than BHT. After storage for 30 days at −20 °C, DMSO solutions of most of the derivatives of 4-NC were more stable and exhibited more antioxidant activity than 4-NC, quercetin and BHA and exhibited comparable antioxidant activity to BHT. These findings point to the potential of derivatives of 4-NC as antioxidant compounds.

## 1. Introduction

**Figure 1:**
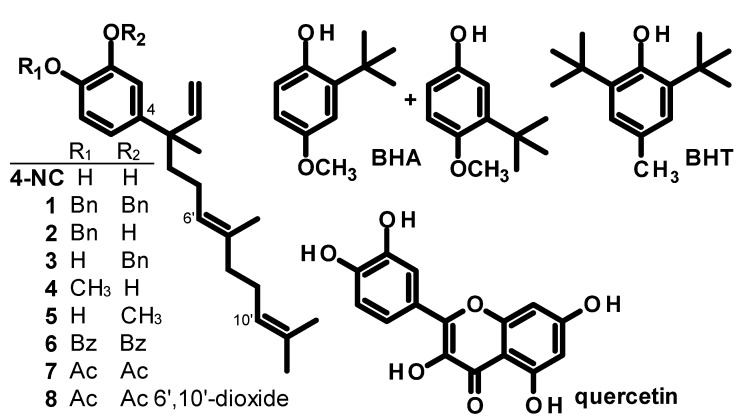
Structures of 4-NC (4-nerolidylcatechol), semi-synthetic derivatives of 4-NC **1**–**8** and synthetic food additives BHA (butylated hydroxyanisole) and BHT (butylated hydroxytoluene) and the flavonoid quercetin.

4-Nerolidylcatechol (4-NC, Figure 1) is a secondary plant metabolite which is found in the roots, leaves and inflorescences of *Piper peltatum* L. and *Piper umbellatum* L. (syn. *Pothomorphe peltata* (L.) Miq. and *Pothomorphe umbellata* (L.) Miq., respectively, Piperaceae family). Interestingly, 4-NC makes up >5% of the dry weight of mature *P. peltatum* roots and is readily isolable on a multi-gram scale through extraction of the roots of *P. peltatum* (or *P. umbellatum*) as the initial step and purification by column chromatography as the final step [1,2]. Agronomic studies conducted near Manaus, Amazonas State, Brazil have demonstrated that *P. peltatum* can be readily cultivated [3,4]. Grown from seed, *P. peltatum* has a life span of approximately 500 days after transplanting (DAT). There is an interplay between root biomass and levels of 4-NC in the root which leads to a maximum in per hectare 4-NC production (*ca*. 27 kg/ha) at around 425 DAT [4]. Thus, 4-NC is available on a large scale through extraction and purification from the roots of naturally occurring or cultivated *P. peltatum* and *P. umbellatum*. The role of 4-NC in *P. peltatum* and *P. umbellatum* metabolism and the relevance of the production of this secondary metabolite to the livelihood of these plant species are not understood.

Independent studies have demonstrated the diverse antioxidant properties of the natural product 4-NC. This compound inhibited hydroxyl radical mediated damage to DNA deoxyribose induced by Fe(II) salts [5,6]. Pasqualoto *et al*. [7] found that the antioxidant activity of 4-NC is associated with the oxidation of the alkenyl side-chain which is also observed in alkenylresorcinols. Also, the electrostatic and lipophilic potentials of the aromatic rings and side chains were calculated. 4-NC exhibited *in vitro* antioxidant activity approximately 20 times that of α-tocopherol (vitamin E) in an assay based on the inhibition of spontaneous lipoperoxidation of rat brain homogenates [8]. Formulations of 4-NC having potential therapeutic and cosmetic applications have been studied. Thus, the stability, solubility and bioavailability of 4-NC (low water solubility) and 2-hydroxypropyl-β-cyclodextrin (HP-β-CD) complexes were studied. It was found that the water solubility of 4-NC increased with increased concentration of HP-β-CD and reached a solubility 13 times that of 4-NC in water [9,10,11].

Independent studies have described the *in vitro* cytotoxic activity of 4-NC. This compound was toxic to *Artemia franciscana* (LC_50_ = 25 ± 3 µM [12]) in the brine shrimp assay [13]. It also inhibited the *in vitro* growth of melanoma cell lines (SK-Mel-28, 103 and 147) and normal fibroblast cells (IC_50_= 20–40 µM and IC_50_ = 50 µM, respectively) [14]. 4-NC induced the accumulation of ubiquinated proteins such as Mcl-1 and acted as a proteasome inhibitor in melanoma cells [15]. It is toxic *in vitro* to KB tumor cells and inhibits human topoisomerase I, which has been suggested to be the mechanism of the cytotoxic action of 4-NC in KB cells [16].

4-NC is a component of *Piper umbellatum* and *P. peltatum* extracts that exhibits important pharmacological activity. Publications on the *in vitro* and *in vivo* antimalarial activity of extracts of *P. peltatum* and *P. umbellatum* prompted us to perform a phytochemical study in which 4-NC was isolated from the roots of *P. peltatum* and shown to exhibit high *in vitro* activity against the human malaria parasite *Plasmodium falciparum* [2] and low *in vivo* activity in *Plasmodium berghei*-infected mice [17]. Also, *P. umbellatum* extracts prevent α-tocopherol depletion after UV-irradiation [18], exhibit a strong photoprotective and anti-inflammatory effect against UV radiation-induced skin damage in hairless mice [19] and exhibit other anti-inflammatory and analgesic effects [20,21]. Further research is necessary to investigate the role of 4-NC (if any) in the pharmacological action of *P. peltatum* and *P. umbellatum* extracts.

Semi-synthetic derivatives **1**–**8** of 4-NC with one or two substituent groups (methyl, acetyl, benzyl, benzoyl) on the O atoms and one *O,O*-disubstituted, side-chain modified derivative of 4-NC have been introduced. These 4-NC derivatives exhibit increased stability compared to 4-NC [1,22,23]. Stability of 4-NC and its derivatives has not been systematically studied. Generally, 4-NC is stable in dry, ground roots stored at room temperature for years. However, the half-life of pure 4-NC or solutions of 4-NC at room temperature is on the order of hours and that of pure 4-NC in the freezer (−20 °C) is on the order of weeks. Pure derivatives of 4-NC have freezer half-lives on the order of years. Some of these derivatives of 4-NC exhibited *in vitro* cytotoxicity (IC_50_ = 15.5–63.1 µM) [1,22] comparable to 4-NC (IC_50_ = 19.7–38.8 µΜ) [1] against tumor cell lines. Thus, the 2*O-*monomethyl 4-NC derivative **5** exhibited cytotoxicity (IC_50_ = 43.9–63.1 µM) to colon tumor cells (HCT-8 strain), central nervous system tumor cells (SF295 strain) and leukemia cells (HL-60 strain) and the 1*O*-monomethyl derivative of 4-NC **4** only inhibited the growth of leukemia cells (HL-60 strain, IC_50_ = 57.4 µM) [22]. The 2*O*-monobenzyl derivative of 4-NC **3** also exhibited cytotoxicity (IC_50_ = 25.1–41.7 µM) against four tumor cell lines [22] and *O,O-*diacetyl-4-nerolidylcatechol (**7**) inhibited (IC_50_ = 15.5–15.6 µM) human leukemia cells (HL-60 and CEM) [1]. *O,O*-Dibenzyl, 1*O*-monobenzyl and *O,O*-dibenzoyl derivatives of 4-NC (compounds **1**, **2** and **6**, respectively) were inactive against tumor cells [22]. The cytotoxicity of derivatives of 4-NC to normal cell lines has not been reported previously.

4-NC derivatives **1**–**8** exhibit significant *in vitro* inhibitory activity against the K1 strain of the human malaria parasite *Plasmodium falciparum* K1 (IC_50_ = 0.67–23 µM) that is comparable to the activity of 4-NC [22,23]. As stated above, 4-NC exhibits little activity *in vivo* against *Plasmodium berghei* [17] whereas the *O,O*-diacetyl derivative of 4-NC (compound **7**, Figure 1) exhibits significant *in vivo* inhibitory activity in *P. berghei*-infected mice at oral doses of 1–10 mg/kg/day (2.5–25 µmol/kg/day) in the 4-day suppressive test [24]. Antioxidant and anti-inflammatory properties may be important for the antiplasmodial mode of action of derivatives of 4-NC. Also, since 4-NC is a relatively abundant natural product its simple derivatives may have potential as novel antioxidant compounds for use in a number of applications, including substitution of BHT and BHA as food additives. Thus, we decided to quantitatively and comparatively investigate the antioxidant properties and stability of the antioxidant effects of derivatives of 4-NC as a means to explore the potential utility of these compounds.

## 2. Results and Discussion

### 2.1. Radical Scavenging and Antioxidant Activities of 4-NC and Semi-Synthetic Derivatives of 4-NC

**Figure 2:**
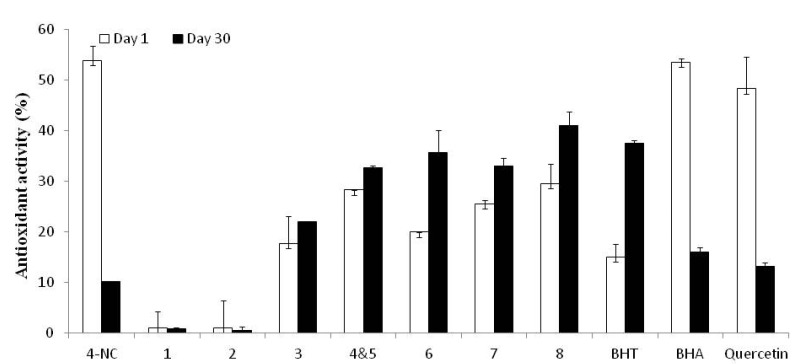
Antioxidant activity of DMSO solutions of 4-NC, 4-NC derivatives and control standards in the 3T3L1 mouse embryonic fibroblast cell line on day 1 and after storage at −20 °C for 30 days.

4-NC exhibited high antioxidant activity as a radical scavenger in the DPPH and ABTS assays. These tests evaluate the reducing effect of a given substance on the radical species formed *in situ* during these assays leading to a decrease in color *versus* controls that is measured spectrophotometrically. In these experiments, after dilution in DMSO, 4-NC and semi-synthetic derivatives **1**–**8** were tested and several of these significantly inhibited the formation of radicals. Thus, at a concentration of 250 µM, 4-NC inhibited DPPH• and ABTS+• by 97% and 95%, respectively. Mono-*O*-substituted 4-NC derivatives **2**–**5** inhibited ABTS+• by 89–93%. These same mono-*O*-substituted 4-NC derivatives were less active in the DPPH assay (41–48% inhibition) than 4-NC and BHA (98%) and more active than BHT (17%). In general, the results for radical scavenging and antioxidant activities of 4-NC and mono-*O*-substituted 4-NC derivatives were comparable to those of commercial antioxidants BHT and BHA commonly used as food preservatives (Table 1). The antioxidant activity was also evaluated in mouse embryonic 3T3L1 fibroblasts. In this assay DCFH-DA (2',7'-dichlorofluorescein diacetate) is hydrolyzed by intracellular esterases and then oxidized by reactive oxygen species (ROS) to the fluorescent compound 2'-7'-DCF. An active antioxidant substance can inhibit the transformation of fatty acids to fatty acid hydroperoxides in the cell membranes and thereby inhibit the oxidation of DCFH-DA. In this assay both 4-NC and several of the 4-NC derivatives were able to penetrate the cell and inhibit oxidation of DCFH-DA thereby demonstrating antioxidant activity in a concentration dependent manner. As shown in Figure 2, 4-NC, a mixture of 4-NC derivatives **4** and **5** and 4-NC derivatives **6**, **7** and **8** individually inhibited 20% or more of the oxidation of DCFH-DA in 3T3L1 cells over the observation period of 60 min. The antioxidant activity of phenols and catechols is very well know and is at least partly explained by the ability of these compounds to chelate metals such as iron and copper which may exhibit oxidizing activities or the ability to stabilize free radicals depending on their chemical structure [25,26].

**Table 1:** Free radical scavenging activity and cytotoxicity of 4-NC, derivatives of 4-NC and commercial antioxidant food additives BHA and BHT.

Compound	ABTS	DPPH•	Toxicity			
			Brine shrimp ( *Artemia franciscana*)		Mouse embryonic fibroblast cells (3T3L1 line)	
	(%) *	(%) *	**(LC50)**	**(IC50, µM)**		
4-NC	94.82 ± 0.22 ^a^	96.53 ± 0.50 ^a^	25 M	31.5 (25.8–38.2)			
**1**	7.97 ± 2.72 ^b^	4.35 ± 0.71 ^c^	>1.0 mM ^†^	>101 ^‡^			
**2**	91.12 ± 0.68 ^a^	44.20 ± 0.01 ^b^	1.0 mM	43.1 (37.9–49.0)			
**3**	93.31 ± 0.30 ^a^	48.09 ± 1.33 ^b^	>1.2 mM ^†^	>124 ^‡^			
**4** & **5**	89.05 ± 0.44 ^a^	40.52 ± 0.51 ^b^	0.60 & 1.2 mM **	>152 ^‡^			
**6**	8.83 ± 0.23 ^b^	3.46 ± 0.63 ^c^	69 µM	>95.8 ^‡^			
**7**	8.60 ± 0.10 ^b^	6.36 ± 1.15 ^c^	83 µM	ND			
**8**	0.31 ± 0.01 ^c^	7.39 ± 3.66 ^c^	ND	>116 ^‡^			
BHT	93.13 ± 0.18 ^a^	16.84 ± 0.81 ^c^	ND	ND			
BHA	94.71 ± 0.47 ^a^	97.97 ± 0.10 ^a^	ND	ND			
Quercetin	99.7 ± 0.02 ^a^	91.4 ± 0.12 ^a^	ND	ND			

* In the ABTS and DPPH assays the compounds were tested at 250 µM and values presented represent mean ± standard deviation. The mean values are based on replicate readings (n = 3). Values with different letters within the same column are significantly different (*p* < 0.05). Statistical analysis was by one-way ANOVA followed by Dunnett’s Multiple Comparison. The number presented represents the percentage of inhibition observed. ** Compounds were tested separately. ^†^ >500 µg/mL. ^‡^ >50 µg/mL.

Quercetin and BHA lost 70% or more of their initially observed antioxidant activity after 30 days, which is expected given that they were stored in solution at low concentration. Surprisingly, the antioxidant activity of BHT in DMSO solution increased approximately 100% after 30 days of storage in the freezer. This is most likely the result of the instability of BHT when in solution and biological factors such as the interactions of BHT decomposition products within the cells. The decomposition products of BHT are well known such as 2,6-di-tert-butyl-p-benzoquinone, 3,5-di-tert-butyl-4-hydroxybenzaldehyde and 3,5-di-tert-butyl-4-hydroperoxy-4-methyl-2,5-cyclohexadien-1-one [27]. Presumably, one or more BHT metabolites is a more effective antioxidant than BHT in this whole-cell model of antioxidant activity.

### 2.2. Cytotoxicity of 4-NC and Derivatives of 4-NC

The cytotoxicity of 4-NC and derivatives of 4-NC **1**–**8** to mouse embryonic fibroblast line 3T3L1 was evaluated using alamar blue to measure cell survival/proliferation. The substances at different concentrations (12.5, 25 and 50 µM) were separately incubated with cells and the number of viable cells was counted after 24, 48 and 72 h. 4-NC exhibited moderate cytotoxicity (IC_50_ = 9.9 µM). Only one derivative of 4-NC, 1-*O*-benzyl 4-NC (**2**), was cytotoxic to mouse embryonic fibroblast cells (IC_50_ = 17.5 µM) (Table 1). All other derivatives of 4-NC exhibited no significant cytotoxicity at the highest concentration tested (50 µM), an indication that these derivatives of 4-NC exhibit low toxicity compared to 4-NC. Mono-*O*-benzyl 4-NC derivatives **2** and **3** and the mono-*O*-methyl 4-NC derivatives **4** and **5** exhibited weak cytotoxic activity against *Artemia franciscana* in the brine shrimp assay and *O,O*-dibenzoyl 4-NC (**6**) and *O,O*-diacetyl 4-NC (**7**) were moderately toxic to brine shrimp. *O,O*-Dibenzyl 4-NC (**1**) was not active (*i.e.* not toxic to *A. franciscana*) in the brine shrimp assay (Table 1).

### 2.3. Antioxidant Activity and Stability of 4-NC and Derivatives of 4-NC

The antioxidant activity of 4-NC and semi-synthetic derivatives of 4-NC was evaluated on the 1st and 30th days after dissolving the substances in DMSO at a concentration of 10 mM and storage in a freezer at −20 °C. This experiment permitted the evaluation of the relative stability of the antioxidant activity of 4-NC, derivatives of 4-NC, BHT and BHA. Thus, cellular antioxidants were evaluated using the mouse embryonic fibroblast 3T3L1 cell line. From the results of this assay, it was evident that some of the derivatives of 4-NC exhibited greater stability in solution than did 4-NC, BHA or BHT. The presence of mono-*O* or di-*O,O* substituent groups in the 4-NC derivatives can confer stability relative to 4-NC. Such chemical stability is highly desirable when contemplating the use of such substances in cosmetic and pharmaceutical formulations and foods. Here, 4-NC dissolved in DMSO lost more than 80% of its antioxidant activity after 30 days in the freezer. Also, significant losses of antioxidant activity were observed after 30 days for DMSO solutions of BHT and BHA. Derivatives of 4-NC that were significantly active on the 1st day did not exhibit lower antioxidant activity after 30 days in DMSO solution at −20 °C thus attesting to high antioxidant activity and high stability of the antioxidant effect of derivatives of 4-NC in DMSO solution over time as compared to 4-NC, BHT and BHA (Figure 2).

### 2.4. Structure-Activity Relationship in 4-NC, Derivatives of 4-NC, BHT and BHA

The structures of 4-NC, derivatives of 4-NC, antioxidant synthetic standards BHA and BHT and antioxidant natural product standard quercetin are presented in Figure 1. These compounds have in common a highly-substituted 6-membered aromatic (benzene) nucleus which is activated by one or two oxygen-containing substituents and substituted by one or two quaternary carbon-containing moieties. There is evidence that catechols (including 4-NC) and polyphenols containing catechol substructures (such as the flavonoid quercetin) provide cytoprotective effects and protect DNA from damage. Further evidence exists that iron chelation plays a role in these effects and that lipophilicity and the ability to cross cell membranes contribute to the antioxidant effect of phenolic compounds in general [25]. The 4-NC derivatives evaluated herein are *O*-substituted or *O,O*-disubstituted and therefore would not be expected to behave as catechols. However, it is reasonable to assume that some substituents, such as acetyl groups, suffer hydrolysis under physiologic conditions and due to the action of specific and non-specific enzymes and tissues. All 4-NC derivatives have a catechol nucleus that is substituted by a quaternary carbon atom. Quaternary substituents like nerolidyl (in 4-NC derivatives) or *tert*-butyl groups (in BHA and BHT) activate the aromatic ring system and render it more prone to oxidation than when these substituents are absent. This activation involves increased stabilization of radical and other electron deficient intermediates which form during oxidation and is the mechanistic basis for the antioxidant effect of these phenol compounds. As discussed above, 4-NC and several derivatives of 4-NC were able to penetrate the cell and inhibit oxidation of DCFH-DA in a concentration dependent manner. The penetration of 4-NC and derivatives of 4-NC into cells and their supposed action in the cell membrane against the oxidation of lipids by reactive oxygen species (ROS) may be related to the highly lipophilic C_15_ terpenoid hydrocarbon side chain present in these compounds and its affinity for membrane phospholipids.

4-NC was the most active antioxidant compound tested in the ABTS and DPPH assays. This is presumably due to the presence of the two catechol OH groups in this natural compound. 4-NC was also the most cytotoxic of all the compounds in this study. In general, *O,O*-disubstituted derivatives of 4-NC exhibited little or no antioxidant/radical scavenging activity. Mono-*O*-substituted derivatives of 4-NC were in general as antioxidant or more antioxidant than BHT and BHA in the ABTS and DPPH assays. Thus, the presence of at least one phenol OH in 4-NC and mono-*O*-substituted derivatives of 4-NC is key to the comparable antioxidant activity of 4-NC, derivatives of 4-NC, BHA and BHT.

## 3. Experimental

### 3.1. Chemicals and Reagents

Dulbecco’s Modified Eagle Medium (DMEM) was purchased from Invitrogen (Carlsbad, CA, USA). Resazurin, 2,2'-azinobis-3-ethylbenzothiazoline-6-sulfonic acid (ABTS), 2,2-diphenyl-1-picrylhydrazyl (DPPH), 2'7'-dichlorofluorescein diacetate (DCF-DA), quercetin, butylated hydroxyanisole (BHA) and butylated hydroxyl toluene (BHT) were purchased from Sigma-Aldrich^®^ (St. Louis, MO, USA).

### 3.2. Derivatives of 4-NC

#### 3.2.1. Cultivation of *Piper peltatum*

*Piper peltatum* L. was cultivated at Embrapa Amazonia Ocidental near the city of Manaus, Amazonas State, Brazil using published procedures [3,4].

#### 3.2.2. Extraction and Isolation of 4-NC

Following established procedures, roots of mature plants were harvested, dried, milled and extracted with solvents. After filtration and evaporation of solvents, extracts were either directly chromatographed on silica gel to yield pure 4-NC [2] or liquid-liquid partitioned to yield a 4-NC-rich fraction which was then chromatographed [1]. Pure 4-NC is labile and is best stored under nitrogen in the freezer (−20 °C).

#### 3.2.3. Preparation of Derivatives from 4-NC and Purity of 4-NC Derivatives

4-NC was the substrate for *O*-methylation, *O*-benzylation, *O*-acetylation and *O*-benzoylation reactions which yielded one or more products in each reaction. After work up, normal phase column chromatography, preparative thin-layer chromatography and other chromatographic techniques were used to separate and/or purify each derivative of 4-NC. Details of the preparations of semi-synthetic derivatives of 4-NC used herein are provided in Pinto *et al*. [1,22] and a patent [23] and NMR, HRMS and other useful data are available for derivatives of 4-NC [1,22]. The purity of derivatives was re-checked immediately prior to assaying using aluminum-backed analytical thin-layer chromatography (TLC) plates (Merck, Darmstadt, Germany) and different solvent systems as eluents and should be considered to be >95%.

### 3.3. Cell Culture

3T3-L1 cells (mouse embryonic fibroblasts) were obtained from the Cell Bank of Rio de Janeiro, Brazil and were maintained in Dulbecco’s Modified Eagle Medium (DMEM) which was supplemented with 10% fetal bovine serum (FBS), penicillin (100 U/mL) and streptomycin (100 U/mL). The cells were incubated at 37 °C in a humidified atmosphere containing 5% of CO_2_.

### 3.4. Antioxidant Activity Chemical Assays

#### 3.4.1. DPPH Radical-Scavenging Activity

The measurement of the scavenger activity of DPPH• was carried out according to the method of Molineux [28] with modifications. Substances at 250 µM were added, at an equal volume, to the methanol solution of DPPH• (100 mM). After 15 min at room temperature, the absorbance was recorded at 517 nm (sample abs). The experiment was repeated three times. BHT, BHA were used as controls. Values denote percentage of DPPH free radical inhibition. The antioxidant activity was calculated using the equation: % inhibition = 100 × (1 − sample abs/control abs).

#### 3.4.2. ABTS (2,2'-Azinobis-3-ethylbenzothiazoline-6-sulfonic acid) Radical-Scavenging Activity

The ABTS assay used herein was based on the method of Re *et al*. [29] with slight modifications. Substances at concentrations of 250 µM, BHT or BHA were added to dilute ABTS solution and the absorbance at 714 nm was measured. The antioxidant activity was calculated using the equation: % inhibition = 100 × (1 − sample abs/control abs).

### 3.5. Cell Viability Assay

The cytotoxicity of 4-NC and its semi-synthetic derivatives **1**–**8** to the 3T3L1 mouse embryonic fibroblast cell line fibroblasts was determined by the alamar blue method as described by Nakayama and co-workers [30]. The alamar blue assay is a colorimetric assay involving the cellular reduction of resazurin to resorufin. Briefly, adherent cells (5 × 10^3^ cells/well) were grown in 96-well tissue culture plates and exposed to substances (50, 25 and 12.5 μM) for 24, 48 and 72 h. After incubation, the alamar blue solution (10 µL of 0.4% alamar blue (resazurin) in PBS) was added and the cells were incubated for 2 h at 37 °C. Fluorescence was measured (excitation at 545 nm and emission at 595 nm) and expressed as a percentage of the cells in the control after background fluorescence was subtracted. The assays were performed in triplicate.

### 3.6. Cellular Antioxidant Activity

Intracellular ROS production was detected using the non-fluorescent cell permeating compound, 2'7'-dichlorofluorescein diacetate (DCF-DA), as described by Wolfe and Liu [31]. DCF-DA is hydrolyzed by intracellular esterases and then oxidized by hydroperoxides into the fluorescent compound, 2'-7'-DCF. 3T3-L1 cells were seeded at a density of 6 × 10^4^ cells/well in a 96-well microplate in 100 μL of growth medium. 24 h after seeding, the growth medium was removed and the wells were washed with PBS. Then, a 10 μM solution of DCFH-DA dissolved in Hank’s buffer (100 μL) was added to wells and incubated for 30 min at 37 °C and 5% CO_2_. The cells were washed first with PBS (100 μL) then EEP (100 µL) and samples having different concentrations were added. The fluorescence was immediately measured at an excitation wavelength of 485 nm and emission wavelength of 520 nm over 60 min at 5 min intervals. Controls with/without DCFH-DA were prepared and subjected to analogous procedures. Quercetin, BHA and BHT were used as positive controls of antioxidant activity.

### 3.7. Statistical Analysis

Results were expressed as the means and standard deviations triplicate/quadruplicate measurements. Differences between groups were assessed by one-way analysis of variance (ANOVA) followed by Dunnett’s Multiple Comparison. The 50% inhibitory concentration (IC_50_) values were obtained by non-linear regressions of concentration–response curves. A value of *p* < 0.05 indicated significance.

## 4. Conclusions

4-NC, mono-*O*-methyl and mono-*O*-benzyl derivatives of 4-NC exhibited comparable antioxidant activities to BHT and BHA in the DPPH and ABTS assays. This is highly relevant given the widespread commercial use of BHT and BHA as preservatives in foods. 4-NC and some of its derivatives significantly inhibited the oxidation of DCFH-DA in the mouse 3T3L1 fibroblast cell line. In general, derivatives of 4-NC were less cytotoxic than 4-NC. Significant declines in the antioxidant activity in the cell model were observed for DMSO solutions of 4-NC, BHT and BHA. This contrasts with derivatives of 4-NC which were initially less active than 4-NC, BHT and BHA, but several derivatives of 4-NC proved to be more stable and maintained significant antioxidant activity to cells after 30 days of storage in DMSO at −20 °C. These preliminary results point to the potential of 4-NC derivatives as potent antioxidant, radical scavengers that are capable of intracellular antioxidant action.
